# Infection of Human Macrophages by *Leishmania infantum* Is Influenced by Ecto-Nucleotidases

**DOI:** 10.3389/fimmu.2017.01954

**Published:** 2018-01-11

**Authors:** Nalu Teixeira de Aguiar Peres, Luana Celina Seraphim Cunha, Meirielly Lima Almeida Barbosa, Márcio Bezerra Santos, Fabrícia Alvise de Oliveira, Amélia Maria Ribeiro de Jesus, Roque Pacheco de Almeida

**Affiliations:** ^1^Laboratory of Molecular Biology, Department of Medicine, University Hospital, Federal University of Sergipe, São Cristóvão, Brazil; ^2^Department of Morphology, Biological and Health Sciences Centre, Federal University of Sergipe, Aracaju, Brazil; ^3^Department of Health Science, Federal University of Sergipe, Aracaju, Brazil; ^4^Instituto de Investigação em Imunologia, Institutos Nacionais de Ciência e Tecnologia, Conselho Nacional de Desenvolvimento Científico e Tecnológico (CNPq), Brasília, Brazil

**Keywords:** *Leishmania infantum*, macrophage, infection, ecto-nucleotidase, extracellular nucleotides

## Abstract

Ecto-nucleotidase activity is involved in the infection process of *Leishmania* and various other parasites that enables modulation of host immune responses to promote disease progression. One of the enzymes responsible for this activity is the ecto-nucleoside triphosphate diphosphohydrolase (E-NTPDase). The enzyme hydrolyzes nucleotides tri- and/or di-phosphate into monophosphate products, which are subsequently hydrolyzed into adenosine. These nucleotides can serve as purinergic signaling molecules involved in diverse cellular processes that govern immune responses. Given the importance of the extracellular metabolism of these nucleotides during intracellular pathogen infections, this study evaluates the role of ecto-nucleotidase activity during *Leishmania infantum* (*L. infantum*) infection in human macrophages. E-NTPDase protein expression and activity was evaluated in *L. infantum* during purine starvation, adenosine-enriched medium, or in the presence of an inhibitor of ecto-nucleotidases. Results show that E-NTPDase is expressed in *L. infantum* parasites, including on the cell membrane. Furthermore, functional activity of the enzyme was modulated according to the availability of adenosine in the medium. Purine starvation increased the hydrolytic capacity of nucleotides leading to higher infectivity, while growth in adenosine-enriched medium led to lower infectivity. Moreover, inhibiting E-NTPDase function decreased *L. infantum* infection in macrophages, suggesting the enzyme may serve as a ligand. Taken together, the ability of *L. infantum* to hydrolyze nucleotides is directly associated with increased infectivity in macrophages.

## Introduction

Visceral leishmaniasis (VL) is a neglected parasitic disease caused by species of the genus *Leishmania*, which frequently leads to death if untreated (1, 2). It is estimated that about 200,000 new cases of VL occur yearly worldwide and that over 90% occur in Bangladesh, Brazil, Ethiopia, India, South Sudan, and Sudan (W.H.O. 2015). VL has a mortality rate of about 5% and has few clinical treatment options (3).

Several factors are determinant for the clinical outcome of VL. Disease severity can range between subclinical and different degree of severity. Several factors involved in early host–parasite interactions and innate immune responses can directly modulate VL severity. The release of nucleotides, as well as the enzymes that participate in the degradation of these nucleotides and their receptors, into the extracellular environment can directly influence early events of host–parasite interactions (4). Extracellular adenosine triphosphate (ATPe) and its metabolites, ADP and adenosine, are important mediators of the immune response (5). Extracellular levels of ATP and adenosine are detected and transduced by the purinergic receptors of type P2 and P1, respectively. Most immune cells express P2 and P1 receptors. Depending on the concentration, ATPe can act as an immunostimulatory molecule, whereas adenosine often triggers immunosuppressive responses (6). Under normal physiological conditions, ATP is almost exclusively present inside the cells at high concentrations (in the millimolar range). In the extracellular environment, ATP concentration is negligible (at low nanomolar ranges) (7). ATP can serve as a potent signal of distress or damage. Cell damage often leads to ATP release, which can be detected by neighboring and surrounding cells. Detection leads to transmission of signals *via* ATP-conducting pathways to produce nucleotidases to degrade the ATPe (8). Interestingly, *Leishmania* sp. is able to modulate the concentration of extracellular nucleotides, thereby altering the balance of pro- and anti-inflammatory molecules to evade host immune responses (9).

Ecto-nucleoside triphosphate diphosphohydrolase (E-NTPDase) enzymes are another important element in the metabolism of nucleotides. The enzymes, also classified in the nucleoside triphosphate diphosphohydrolases family of ecto-nucleotidases or apyrases, are important for parasite nutrition by facilitating acquisition of extracellular purines. E-NTPDase has been suggested to play important roles in host–parasite interaction and has been identified as being a virulence factor that participates in adhesion and infection in trypanosomatides (10–13). Studies have further shown an association between ATP hydrolysis and the biological effects in host–parasite interaction (14–18).

The activity of ecto-nucleotidases has been demonstrated in *Leishmania tropica, Leishmania amazonensis*, and *L. infantum* (12, 19, 20). These enzymes play a role in virulence, cell adhesion, parasite release from infected cells, and in control of nucleotide concentrations inside cells and in the extracellular spaces (17, 21, 22). Moreover, these enzymes also plays a role in the immune response by favoring reduction of IFN -γ and increased IL-10 expression, thus dampening the host immune response to facilitate the parasite’s survival (11, 22–24).

Purine starvation increases ecto-nucleotidase activity, thereby suggesting a major importance of these enzymes for parasite nutrition and survival. It is believed that the role of ecto-nucleotidases during infection is to degrade nucleotides molecules in the extracellular environment in support of parasitic infectivity and reproduction. However, the effect of purine starvation, as well as the supplementation of adenosine to the parasites during macrophage infection, has not been investigated. Furthermore, it is unknown whether these enzymes, present in the *Leishmania* membrane, can also facilitate parasite entry into host macrophages. Therefore, this study aimed to evaluate the influence of E-NTPDase activity during *L. infantum* infection in human macrophages. Moreover, as these early events of infection could influence the disease severity, we also aimed to compare the levels of E-NTPDase activity in different clinical isolates from VL patients.

## Materials and Methods

### Parasites

*Leishmania infantum* strains LVHSE09, LVHSE17, LVHSE23, and LVHSE49 were obtained from bone marrow aspirates of visceral leishmaniasis patients, as previously reported (25). The promastigotes were cultured in Schneider’s insect medium (Sigma Aldrich) pH 7.2, supplemented with 10% heat-inactivated fetal bovine serum (FBS) and 100 U/ml of penicillin/streptomycin.

### Detection of *L. infantum* E-NTPDase-1 and E-NTPDase-2 by Confocal Microscopy

For the immunolocalization of E-NTPDase-1 and E-NTPDase-2 in *L. infantum*, promastigotes were washed in PBS and fixed in 0.1% glutaraldehyde and 2% paraformaldehyde in 0.1% cacodilate. The parasites were then placed onto glass slides containing 1% poly-L-lysine (Sigma Aldrich). Parasites were treated with 50 µM glycine for 30 min prior to blocking with 1% BSA containing 0.01% Tween 20 in PBS (PBS-T BSA). Samples were incubated with anti-human E-NTPDase 1 or 2 antibodies or respective isotype controls (Sigma Aldrich) in a 1:20 dilution for 12 h. Slides were then washed three times with PBS-T BSA, and incubated with secondary anti-rabbit IgG antibody conjugated to Alexa 488 (Santa Cruz Biotechnology) at a 1:100 dilution for 2 h. Slides were mounted and images acquired using Leica^®^ SP8 confocal microscope.

### Western Blot of the E-NTPDase-1 and 2 in Total Extract of *L. infantum*

Western blot studies were performed as previously described (12). Briefly, total extract of *L. infantum* was obtained after growth of 1 × 10^8^ cells/ml until stationary phase. Cells were lysed using NP-40 lysis buffer supplemented with a protease inhibitor cocktail (2 mM AEBSF, 0.3 µM Aprotinina, 116 µM Bestatina, 14 µM E-64, 1 µM Leupeptina, and 1 mM EDTA; Sigma Adrich). After electrophoresis in a SDS-PAGE gel, samples were transferred to nitrocellulose membrane using the mini transblot electrophoretic transfer cell (Bio-Rad). Next, the membrane was blocked with 1% BSA prior to extensive washing in PBS Tween 0.1% (PBS-T). The blot was then incubated overnight with each respective primary antibody at a dilution of 1:250. Membranes were washed three times with PBS-T for 5 min and incubated with respective secondary antibodies conjugated to Alexa 488 (Santa Cruz Biotechnology). After washing three times with PBS-T for 5 min, the membranes were analyzed in the ChemiDoc MP (Bio-Rad).

### *Leishmania* Culture Conditions and Pretreatments

*Leishmania infantum* promastigotes were grown to the stationary phase and then incubated for 1 h with 250 µM suramin (Sigma Aldrich), an inhibitor of the ecto-nucleotidase activity (20). After incubation, promastigotes were washed twice and resuspended in saline solution (0.9% NaCl). To demonstrate that suramin had no effect on parasite growth, promastigotes at a concentration of 3 × 10^5^ cells/ml were grown in the absence or presence of 500 µM suramin. Growth was estimated daily by standard cell counting using a Neubauer chamber. Analysis was performed by two independent observers.

In order to evaluate the activity of ecto-nucleotidases of the parasites under purine starvation, promastigotes were cultured for 48 h in Modified Eagle Medium depleted of purine (300 mg/L l-proline, 14.250 mg/L HEPES, 1 mg/L d-biotin, 0.2 mg/L ascorbic acid, 0.2 mg/L Vitamin B12, 15 mg/L bovine albumin, 10 mg/L phenol red, 0.4 mg/L lipolic acid 0.4 mg/L Menadione, 0.4 mg/L Vitamin A, 10 mg/L Hemin, and 11 mg/L folic acid; pH 7.4). In addition, to evaluate the ecto-nucleotidases activity in purine-enriched environment, promastigotes were grown in medium supplemented with 200 µM adenosine. After each culture condition, the ecto-nucleotidase activity was measured and the parasites were used to infect macrophages. To evaluate whether the human anti-E-NTPDase-1 blocking antibody inhibits E-NTPDase activity, promastigotes were incubated for 1 h in presence of the blocking antibody (diluted at 1:100) prior to measurement of E-NTPDase activity or for use in macrophage infection experiments.

### Hydrolytic Activity of the Ecto-Nucleotidase

Four different strains of *L. infantum* were evaluated for E-NTPDase activity, but only strain LVHSE49 was used for infection studies. Hydrolysis of nucleotides were measured by incubation of *Leishmania* parasites for 1 h in the presence of 5 mM ATP, ADP, or AMP (Sigma Aldrich) in a solution containing 116 mM NaCl, 5.4 mM KCl, 5.5 mM d-glucose, 5 mM MgCl2, and 50 mM Hepes–Tris buffer, as previously described (21). The reaction was started by adding *L. infantum* promastigotes and terminated by the addition of ice cold 0.2 M HCl (10). Non-specific nucleotide hydrolysis was determined by adding the parasites after the reaction was terminated. The suspensions were pelleted and aliquots of the supernatant were used for the measurement of released inorganic phosphate (Pi) (26). Enzymatic activities were expressed as nmol of Pi released. Parasites viability and motility were assessed by trypan blue staining and microscopy (27).

### Infection of Human Macrophages with *L. infantum*

Macrophages derived from peripheral blood mononuclear cells (PBMC) were obtained from healthy donor blood as previously described by Santos et al. (25). Briefly, heparinized venous blood was separated by Ficoll–Hypaque gradient (Sigma Aldrich) to isolate PBMC. The cells were washed twice, counted and resuspended in RPMI 1640 (Sigma Aldrich) supplemented with 10% fetal bovine serum (FBS) prior to plating on Lab-Tek glass plates (Thermo Scientific) at 3 × 10^5^ cells/well. The cells were allowed to adhere for 3 h at 37°C in 5% CO_2_. Non-adherent cells were removed by extensive washing with phosphate-buffered saline (PBS). Adherent monocytes were incubated in RPMI 1640 medium supplemented with 10% FBS at 37°C for 7 days to allow differentiation into macrophages. Next, these macrophages were infected with stationary-phase *L. infantum* promastigotes at ratio of 1:10, respectively. In some experiments, the culture of macrophages with *L. infantum* promastigotes was supplemented with 100 µM ATP to evaluate the role of nucleotide presence influencing infectivity. Extracellular parasites (that did not infect the macrophages) were removed after 2 h by extensive washing. Next, the culture was incubated for an additional 2 h at 37°C. The percentage of infected macrophages and the number of amastigotes per 100 macrophages was analyzed by microscopy. Each set of analyses was performed by two independent observers, with each counting different fields of the slide.

### Statistical Analysis and Ethical Considerations

All experiments were performed in triplicate wells at a minimum of four independent experiments. Statistical analyses were performed using GraphPad Prism 5.0 software (La Jolla, CA, USA) and normality was analyzed by the D’Agostino & Pearson test. Non-parametric data were analyzed using the Mann–Whitney test. Data were considered statistically significant if *p*-value <0.05. Data were expressed as means ± SDs. This project was approved by the Ethical Committee of the Federal University of Sergipe (CONEP), CAAE-0151.0.107.000-07.

## Results

### Ecto-Nucleotidase Activity Does Not Differ among *L. infantum* Isolates

**Figure 1:**
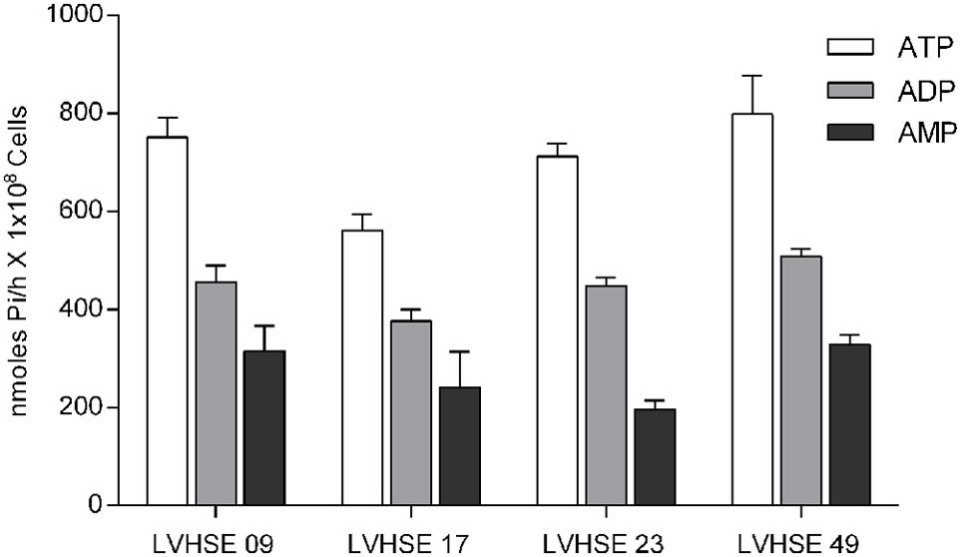
Ecto-nucleoside triphosphate diphosphohydrolase activity of *L. infatum* isolates. Ecto-Nucleotidase activity of *L. infantum* strains LVHSE09, LVHSE17, LVHSE23, and LVHSE49 grown until stationary phase. Promastigotes (1 × 10^8^) were incubated for 1 h at 37°C with ATP, ADP, or AMP, and enzymatic activity was evaluated by measuring inorganic phosphate (Pi) in the extracellular medium. Bars represent the mean ± SD of three independent experiments performed in triplicate.

**Figure 2:**
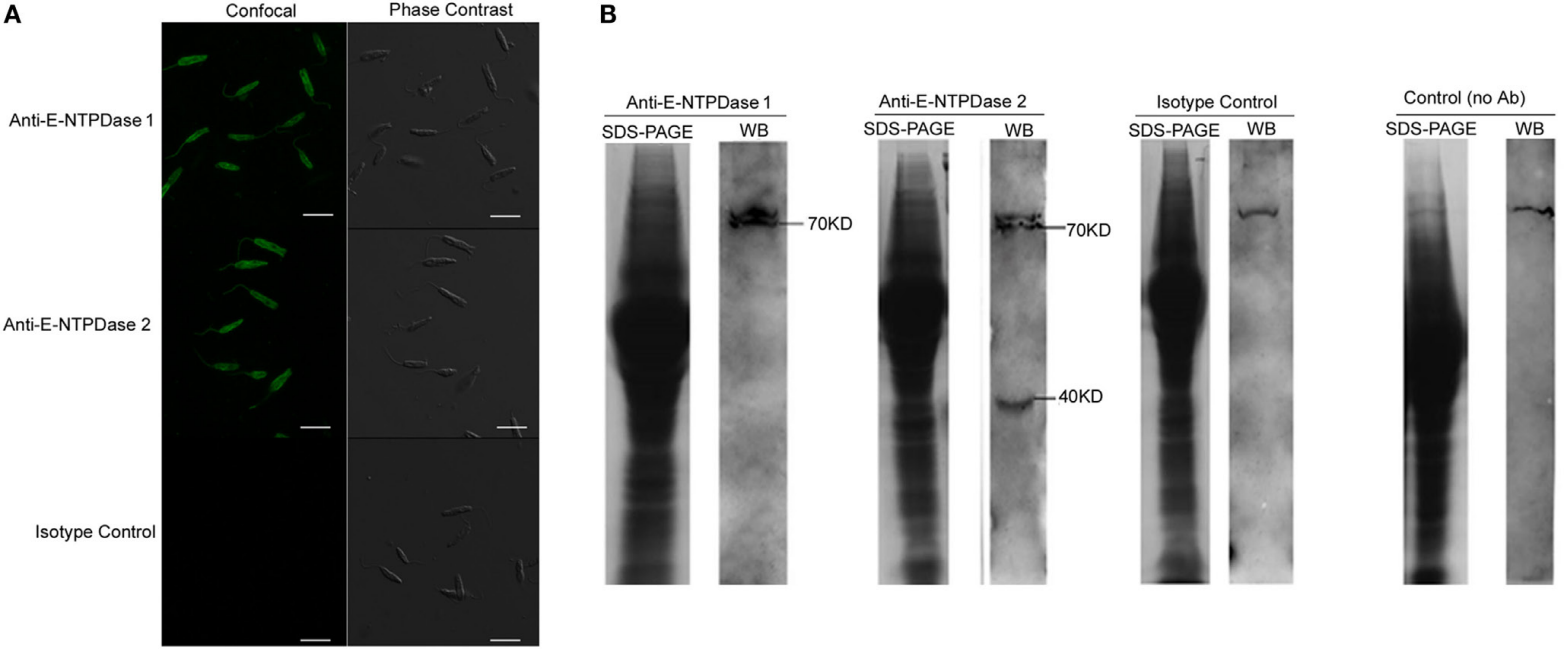
Immunolocalization of ecto-nucleoside triphosphate diphosphohydrolase (E-NTPDase) in *Leishmania infantum*. **(A)** Confocal microscopy showing the presence of E-NTPDase 1 and E-NTPDase 2 in *L. infantum* promastigotes. Parasites were fixed and labeled with primary rabbit antibody IgG anti E-NTPDase 1, anti E -NTPDase 2, or respective isotype IgG controls, followed by secondary labeling with anti-rabbit IgG linked to Alexa 488. **(B)** Western blot of the total extract from *L. infantum* labeled with antibody anti E-NTPDase 1, anti E-NTPDase 2, isotype control, and without antibody. Bars represent 10 µm.

Ecto-nucleoside triphosphate diphosphohydrolase activity was measured and compared among four *L. infantum* strains isolated from different patients. These strains displayed different susceptibility to Nitric Oxide, as previously shown by Santos et al. (25). However, the isolates LVHSE 09, 17, 23, and 49 each did not present significant differences in E-NTPDase activity (Figure 1). Microscopy studies showed that E-NTPDase was present on *L. infantum* promastigotes, including in cellular membrane, as detected by immunostaining with the E-NTPDase 1 and E-NTPDase 2 antibodies (Figure 2A); isotype IgG antibody staining served as an internal control. Interestingly, a homogeneous distribution of E-NTPDase was found throughout the parasite, and also in the cellular membrane. Western blot analysis of the total extract of *L. infantum* promastigotes revealed the presence of two isoforms. A 70 kDa band represented E-NTPDase-1 and the 40 kDa was the E-NTPDase-2 (Figure 2B). The presence of a signal at just above the 70 kDa band was determined to be a non-specific auto-fluorescent signal, as detected in the internal controls (which used no antibodies).

### Ecto-Nucleotidase Expression and Activity in *L. infantum* Influences Infectivity in Human Macrophages

**Figure 3:**
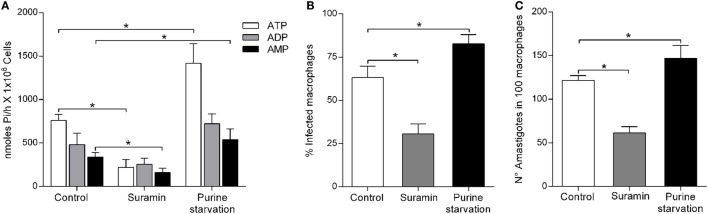
*Leishmania infantum* ecto-nucleoside triphosphate diphosphohydrolase (E-NTPDase) activity and macrophage infection during purine starvation and inhibition by suramin. **(A)** *L. infantum* E-NTPDase activity was evaluated by measuring the inorganic phosphate (Pi) in the extracellular medium of *L. infantum* promastigotes incubated with suramin, or under purine starvation, for 1 h. **(B)** Percentage of infected macrophages after 48 h. **(C)** Number of amastigotes per 100 macrophages after 48 h of incubation. Bars represent the mean ± SD of four independent experiments performed in triplicate.

The ecto-nucleotidase activity of the parasites in the presence of suramin, an inhibitor of protein tyrosine phosphatases, yielded a 70 and 53% reduction in ATP and AMP hydrolysis, respectively, compared to controls (Figure 3A). However, parasites grown under purine starvation resulted in increased hydrolytic capacity, with 65 and 63% for ATP and AMP, respectively (Figure 3A). Interestingly, there was no significant difference in the hydrolysis of ADP.

To assess whether nucleotide hydrolysis is related to the infectivity of *L. infantum*, macrophages infection studies were performed with parasites pretreated with suramin or, alternatively, grown under purine starvation. The infection rate of macrophages with parasites pretreated with suramin was 28.16 ± 3.75%, whereas it was 61.45 ± 5.1% for macrophages infected with untreated control parasites (Figure 3B). In the presence of suramin, the number of amastigotes/100 macrophages was 70.02 ± 4.2 compared to controls at 118.5 ± 5.03 (Figure 3C). No difference was observed in the *L. infantum* growth curve in the presence of suramin (data not shown). Promastigote integrity and viability were confirmed by performing cell motility assays. The number of macrophages infected with parasites treated with the E-NTPDase inhibitor was also about 50% lower in comparison to the control. In addition, the inhibitor treatment resulted in decreased number of intracellular parasites. However, for parasites cultured under purine starvation, the number of infected macrophages was significantly higher (82.75 ± 5.2%) compared to the control (63.16 ± 6.6%) (Figure 3B), as well as the number of amastigotes per 100 macrophages (Figure 3C).

**Figure 4:**
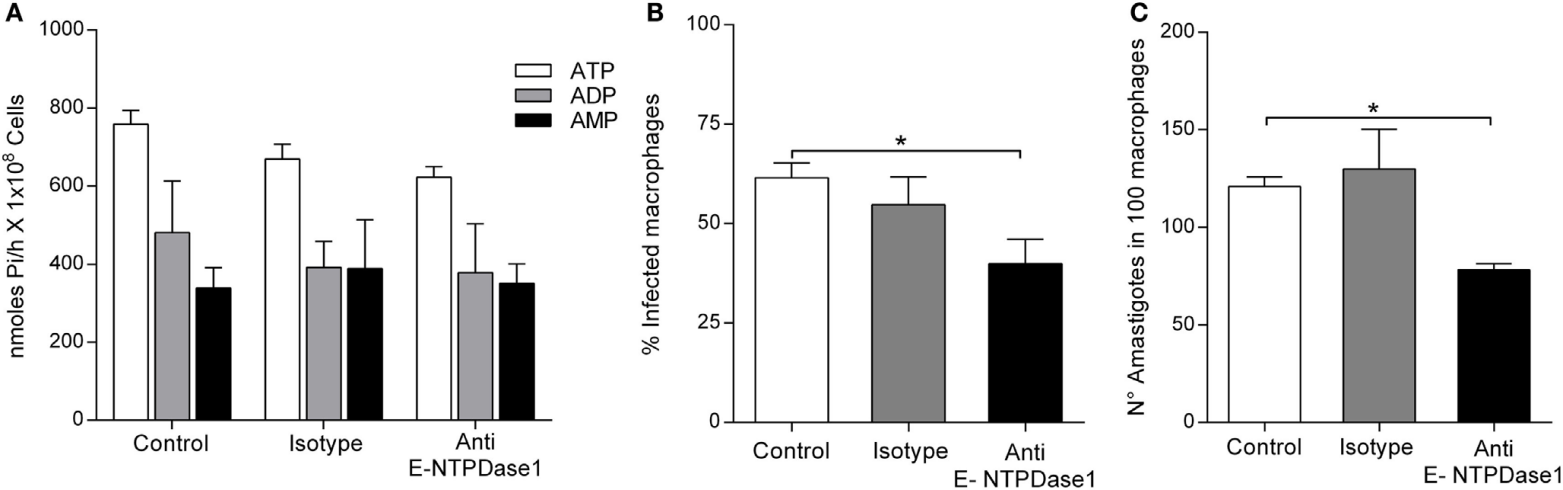
Effect of the blockage of *Leishmania infantum* ecto-nucleoside triphosphate diphosphohydrolase (E-NTPDase) activity and in macrophage infection. **(A)** *L. infantum* E-NTPDase activity was evaluated by measuring the inorganic phosphate (Pi) in the extracellular medium of *L. infantum* promastigotes incubated with anti-human E-NTPDase 1 antibody, for 1 h. **(B)** Percentage of infected macrophages after 48 h. **(C)** Number of amastigotes in 100 macrophages after 48 h. Bars represent the mean ± SD of four independent experiments performed in triplicate, *p < 0.05.

When *L. infantum* parasites were cultured in the presence of E-NTPDase-1 neutralizing antibody, no inhibition of ecto-nucleotidase activity was measured (Figure 4A). However, blocking did reduce macrophage infection rate to 39.9 ± 6.0% compared to 61.4 ± 3.7% in the isotype and untreated controls (Figure 4B). The number of amastigotes per 100 macrophages was also decreased upon addition of the blocking antibody (78.2 ± 3.1) compared to isotype control (120.7 ± 5.0) and untreated control groups (Figure 4C).

### Infection of Human Macrophages by *L. infantum* Is Enhanced by ATP Hydrolysis But Is Decreased When Adenosine Is Available to the Parasite

**Figure 5:**
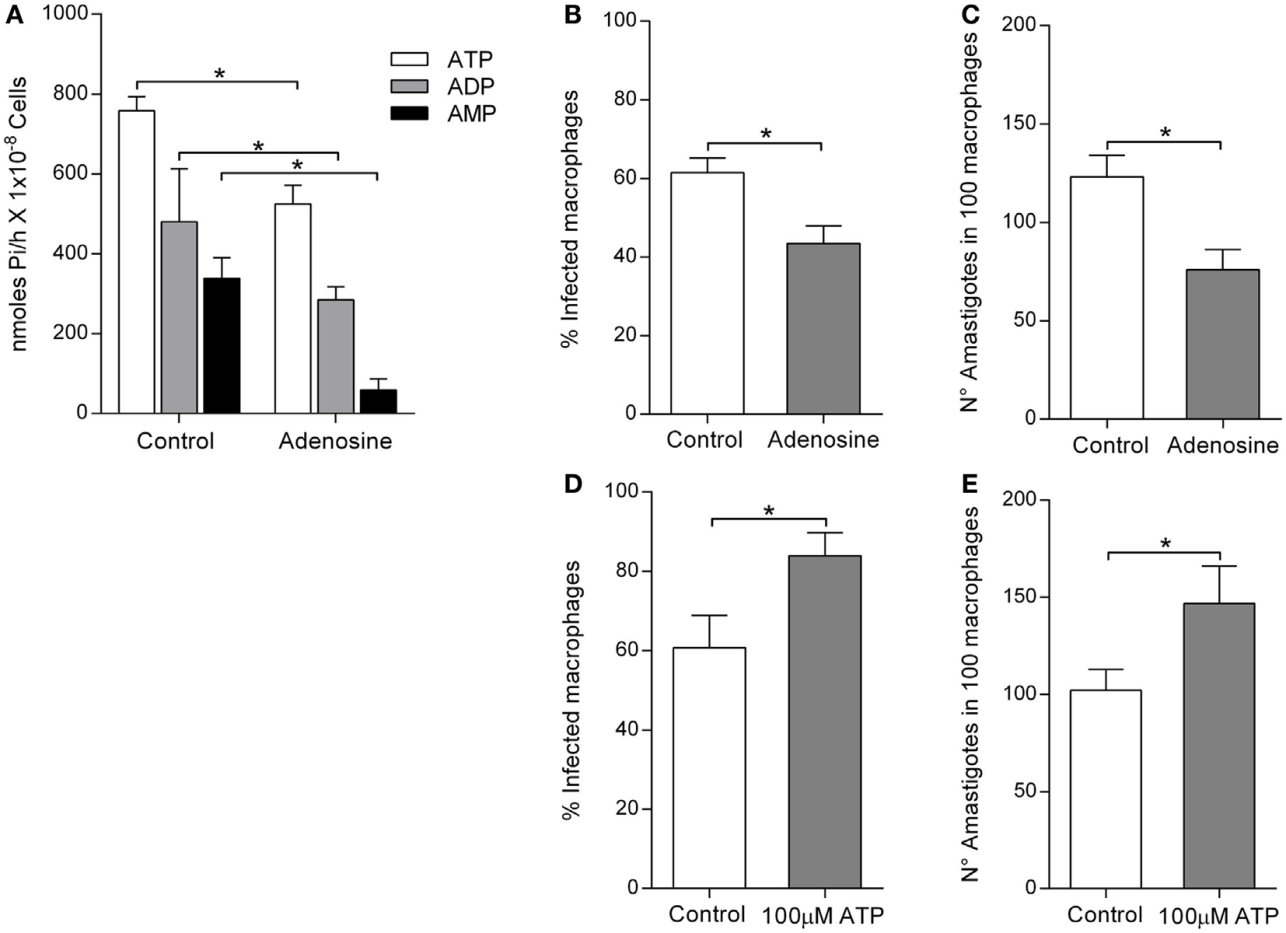
*Leishmania infantum* ecto-nucleoside triphosphate diphosphohydrolase (E-NTPDase) activity and macrophage infection in the presence of adenosine or ATP. **(A)** *L. infantum* E-NTPDase activity was evaluated by measuring the inorganic phosphate (Pi) in the extracellular medium of *L. infantum* promastigotes incubated with adenosine, for 1 h. **(B)** Percentage of infected macrophages after 48 h in the presence of adenosine. **(C)** Number of amastigotes in 100 macrophages after 48 h in the presence of adenosine. **(D)** Percentage of infected macrophages after 48 h in the presence of ATP. **(E)** Number of amastigotes in 100 macrophages after 48 h in the presence of ATP. Bars represent the mean ± SD of four independent experiments performed in triplicate, *p < 0.05.

The purine adenosine is a source for trypanosomatids nutrition. Therefore, we evaluated whether supply of adenosine could alter the nucleotide hydrolysis capacity of *L. infantum* and its ability to infect macrophages. Culture of the *L. infantum* parasites with adenosine decreased ATP, ADP, and AMP hydrolytic capacities when compared to controls (Figure 5A). Moreover, decreased ecto-nucleotidase activity of the parasites resulted in a 30% decrease in the infection rate (43.51 ± 4.3%), compared to the control (61.45 ± 3.7%) (Figure 5B). The number of amastigotes per 100 macrophages was also significantly lower in parasites exposed to adenosine (75.83 ± 10.3) when compared to controls (123.16 ± 13.9) (Figure 5C). In turn, the presence of ATP during infection led to a 25% increase in the infection rate (83.72 ± 5.8%) compared to the control (60.71 ± 8.18%) (Figure 5D). In tandem, the number of amastigotes in 100 macrophages (146.81 ± 19.22) was also increased in the presence of ATP compared to the control (102.16 ± 10.63) (Figure 5E).

## Discussion

This study now shows that high ecto-nucleotidase activity in *L. infantum* parasites correlates directly with a high capacity to infect human macrophages. Furthermore, results revealed that the expression of the enzyme plays an important role in modulating infectivity, independently from the enzymatic activity. Moreover, parasites exposed to purine starvation enhanced nucleotidase activity and infectivity. However, while generation of adenosine by the parasite favors parasite infectivity, pre-exposure to adenosine during parasite growth resulted in increased nucleotidase activity. This demonstrates the importance of ecto-nucleotidase enzymes for the hydrolysis of extracellular nucleotides to modulate infectivity during host–parasite interactions.

The purinergic network signaling of immune cells senses small changes in the concentration of extracellular nucleotides. These responses play multiple roles in immunoregulation by stimulating lymphocytes proliferation, ROS and NO generation, and cytokines and chemokine secretion (9, 28, 29). This study now shows that *L. infantum* exerts NTPDase activity. Although different strains of *L. infantum* have different abilities to overcome NO responses in phagocytes, they do not have significant differences in their ecto-nucleotidase activity. Moreover, imaging studies revealed that these enzymes are present on *L. infantum* and western blot analyses revealed two different isoforms present at 40 and 70 kDa.

Incubation of parasites with suramin inhibited NTPDase activity for ATP, ADP, and AMP. Furthermore, this was followed by a 50% reduction in the infection rate and in the number of amastigote present within macrophages in the infection groups. These results support the notion that E-NTPDase plays an important role in the interaction between *L. infantum* and human macrophages. Previously published studies have shown that increased capacity to hydrolyze extracellular nucleotides is associated with virulence and infectivity of *Leishmania* sp. (22, 30, 31). Furthermore, similar to our findings, inhibition of NTPDase in *T. cruzi* led to a 50% reduction of *in vitro* infection (17). Recognition of *Leishmania* sp. by TLR in phagocytes may trigger the release of ATPe, which binds to the purinergic receptor P2X7. This then leads to activation of the NLRP3 inflammasome pathway and secretion of pro-inflammatory cytokines, such as interleukin-1β (18, 32).

Alternatively, purine starvation led to a higher capacity to hydrolyze adenine nucleotides by the parasites, which also resulted in an increased infection rate. Reports have shown that E-NTPDase activity generates adenosine in the extracellular medium, which allows binding to A2B receptors. This interaction then leads to decreased production of IL-12 and TNF-α by activated macrophages, thereby inhibiting NO production and favoring infection (24). Moreover, deprivation of purines upregulates the expression of enzymes involved in extracellular nucleotide metabolism in attempt to adapt to environmental changes (33–35). In addition, nucleosides can trigger metacyclogenesis in *L. amazonensis*, both *in vivo* and *in vitro* (36). Thus, it is possible that the higher infective capacity of *L. infantum* cultured in media depleted of purine leads to induced metacyclogenesis. However, additional studies are required to understand the effect of purine starvation in the parasites’ life cycle.

In turn, supplementation of adenosine in the culture medium during the *L. infantum* growth significantly decreased NTPDase activity. This is possibly due to downregulation of enzyme expression, potentially because excess adenosine can facilitate the uptake of purines. Moreover, adenosine supplementation led to about 30% reduction in infectivity, corroborating the idea that ecto-nucleotidase activity plays an important role in host–parasite interactions during infections. In *L. amazonensis*, there is a decrease in the E-NTPDase expression when parasites were grown in medium supplemented with adenosine. This led to a reduced survival rate in murine macrophages (24).

However, adenosine often triggers potent immunosuppressive responses by interaction with its P1 receptor on macrophages during infection (6), while ATPe can act in an immunostimulatory capacity. Therefore, *Leishmania* sp. is able to modulate the concentration of extracellular nucleotides, affecting the balance of pro- vs. anti-inflammatory molecules, and thus allowing evasion of host immune responses (9). For example, the blockage of the purinergic adenosine receptor causes decreased lesion size and parasitism in tegumentary leishmaniais (30).

Although the physiological roles of E-NTPDases are not fully understood, some functions have been postulated, such as participation in cell–cell adhesion (21, 37). The adherence of the parasite to host cells is an energy-dependent process (38). We demonstrated that E-NTPDase is expressed in *L. infantum*, including in the cell surface, and its activity promotes infection. The use of neutralizing antibodies to E-NTPDase did not alter the activity of the enzyme, but significantly decreased parasite infectivity in macrophages. This would suggest that not only is its activity important in establishing an infection, but also its localization on the surface of the parasites might favor adhesion to phagocytes. In *T. cruzi* infection, other studies have shown that adhesion and internalization rate is reduced when the NTPDase activity is inhibited by suramin (21). It has also been observed by others that reduction in the adhesion of *L. infantum* to murine macrophages previously treated with recombinant rLicE-2-NTPDase (12).

In conclusion, these studies demonstrate the presence of ecto-nucleotidase activity in human isolates of *L. infantum*. The enzyme is expressed in the parasite, including in the cell membrane, and its nucleotidase activity is increased by purine starvation, which suggests an essential role in parasite survival. Moreover, the ecto-nucleotidase activity of *L. infantum* was shown to be directly associated with infectivity in human macrophages. Antibodies that do not block the enzyme activity resulted in reduced human macrophage infection, suggesting expressed protein is used for infection. In this context, these enzymes, originally known for involvement in the metabolism of nucleotides, are crucial for both parasite nutrition and host–parasite interactions governing infectivity. Taken together, pharmaceutical agents targeting enzyme inhibition could represent novel therapeutic approaches against Leishmaniasis and other trypanosomatides infections.

## Ethics Statement

This project was approved by the Ethical Committee of the Federal University of Sergipe (CONEP), CAAE-0151.0.107.000-07.

## Author Contributions

NP and LC share first authorship. Both performed the majority of the experiments, analyzed datasets, and wrote the manuscript. MA, MS, and FO helped with the macrophage infection experiments. AJ helped to design the experiments and write the manuscript. RA was responsible for experimental design, data analyses, and manuscript revisions.

## Conflict of Interest Statement

The authors declare that the research was conducted in the absence of any commercial or financial relationships that could be construed as a potential conflict of interest.
